# Ion-scale spectral break of solar wind turbulence at high and low beta

**DOI:** 10.1002/2014GL062009

**Published:** 2014-11-25

**Authors:** C H K Chen, L Leung, S Boldyrev, B A Maruca, S D Bale

**Affiliations:** 1 Department of Physics, Imperial College LondonLondon, UK; 2 Space Sciences Laboratory, University of CaliforniaBerkeley, California, USA; 3 Department of Physics, University of Wisconsin-MadisonMadison, Wisconsin, USA; 4 Department of Physics, University of CaliforniaBerkeley, California, USA

## Abstract

The power spectrum of magnetic fluctuations in the solar wind at 1 AU displays a break between two power laws in the range of spacecraft-frame frequencies 0.1 to 1 Hz. These frequencies correspond to spatial scales in the plasma frame near the proton gyroradius *ρ*_i_ and proton inertial length *d*_i_. At 1 AU it is difficult to determine which of these is associated with the break, since 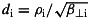 and the perpendicular ion plasma beta is typically *β*_⊥i_∼1. To address this, several exceptional intervals with *β*_⊥i_≪1 and *β*_⊥i_≫1 were investigated, during which these scales were well separated. It was found that for *β*_⊥i_≪1 the break occurs at *d*_i_ and for *β*_⊥i_≫1 at *ρ*_i_, i.e., the larger of the two scales. Possible explanations for these results are discussed, including Alfvén wave dispersion, damping, and current sheets.

## 1. Introduction

The spectrum of magnetic fluctuations in the solar wind forms a power law over several decades, which is thought to be the inertial range of a turbulent cascade [*Alexandrova et al.*, 2013; *Bruno and Carbone*, 2013]. It has long been known that at spacecraft-frame frequencies *f*_sc_∼1 Hz, corresponding to spatial scales in the plasma frame around ion kinetic scales, the spectrum steepens [e.g., *Coleman*, 1968; *Russell*, 1972], although the reason for this remains under debate. At these scales, the turbulent energy is thought to begin to be dissipated, so understanding the spectrum here is crucial to understanding turbulence and heating in collisionless plasmas. In this letter, we present new measurements to investigate the scale associated with the steepening and therefore its cause.

Several ion kinetic scales, related to different physical processes, have been associated with the spectral break, most notably the ion gyroradius *ρ*_i_ and ion inertial length *d*_i_ (see Appendix A for definitions). For example, *Schekochihin et al.* [2009] proposed that the break occurs at the transition between Alfvénic turbulence and kinetic Alfvén turbulence, which would happen when the perpendicular scales become comparable to the ion gyroradius, *k*_⊥_*ρ*_i_∼1, under typical solar wind conditions. The framework of incompressible Hall MHD has also been used to explain the break, in which it occurs at *k*_⊥_*d*_i_∼1 [*Galtier*, 2006], and *d*_i_ has also been suggested to be relevant as the thickness of current sheets which form in the turbulence [*Leamon et al.*, 2000; *Dmitruk et al.*, 2004]. Alternative scales have also been suggested: the wave number at which parallel Alfvén waves are cyclotron damped *k*_c_=*Ω*_i_/(*v*_A_+*v*_th,i_) [*Leamon et al.*, 1998a; *Bruno and Trenchi*, 2014], Landau damping of kinetic Alfvén waves at *kρ*_i_∼1 [*Leamon et al.*, 1999], and when 2*πf*_sc_ becomes comparable to the ion cyclotron frequency *Ω*_i_ [*Denskat et al.*, 1983; *Goldstein et al.*, 1994].

The difficulty in distinguishing these scales with in situ spacecraft observations at 1 AU is that they typically occur at similar spacecraft-frame frequencies. In particular, because 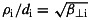, and typically *β*_⊥i_∼1, *ρ*_i_ and *d*_i_ are usually not measurably different. Despite this, there have been several attempts to distinguish between the different scales associated with the break. *Leamon et al.* [1998a] looked at the correlation between the measured break and *k*_c_, found that it was poor and concluded that cyclotron damping was not the cause. *Leamon et al.* [2000] compared three scales—*k*_c_, *d*_i_, and *Ω*_i_—and, under the assumption of oblique propagation, found the best correlation for *d*_i_. *Smith et al.* [2001] examined an interval in which *β*_i_ dropped significantly and noted that the break in the spectrum moved to lower frequencies, as expected for *kd*_i_∼1, rather than *kρ*_i_∼1. A large statistical study was carried out by *Markovskii et al.* [2008], in which the measured break was compared to various theoretical scales. A slightly better correlation was obtained for a combination of scale and fluctuation amplitude, although all theoretical scales displayed moderate correlation, making the results somewhat inconclusive.

An alternative approach has been to use the radial variation of the turbulence to investigate the break. *Perri et al.* [2010] measured the frequency of the break to be independent of distance from the Sun from 0.3 to 4.9 AU, in apparent contradiction to any of the theoretical scales. *Bourouaine et al.* [2012] also measured no significant change in the break frequency from 0.3 to 0.9 AU but concluded that this could be consistent with a break at *k*_⊥_*d*_i_∼1 if the turbulence is highly anisotropic (*k*_⊥_≫*k*_∥_). Most recently, *Bruno and Trenchi* [2014], using higher-resolution data within fast streams, instead found the break frequency to decrease almost linearly with distance from 0.42 to 5.3 AU, similar to 1/*ρ*_i_, 1/*d*_i_, and *k*_c_. The value was found to be closer to *k*_c_, so it was concluded that cyclotron damping must be active.

In this letter, we present a different approach, using several intervals of both *β*_⊥i_≪1 and *β*_⊥i_≫1 in which at least two of the relevant scales, *ρ*_i_ and *d*_i_, are well separated, so that the difference between them is measurable and therefore physically meaningful. The results are then compared to various theoretical predictions, to investigate the cause of the spectral steepening.

## 2. Measurements

**Figure 1:**
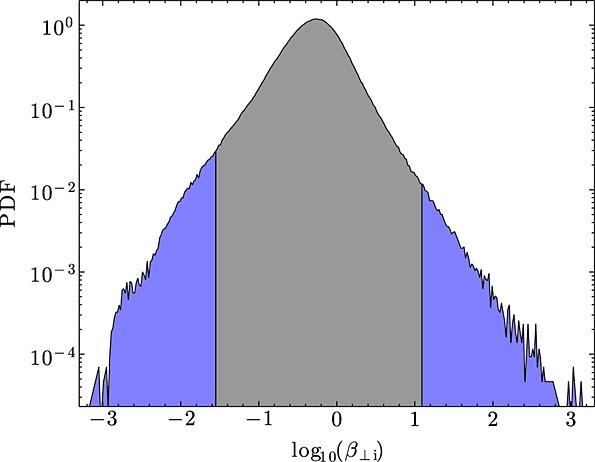
Probability density function (PDF) of log_10_(*β*_⊥i_) in the solar wind. Intervals used in this letter are from the extreme parts of the distribution with *β*_⊥i_≪1 and *β*_⊥i_≫1 shaded in blue.

The solar wind typically has *β*_⊥i_∼1 and only rarely contains periods of *β*_⊥i_≪1 and *β*_⊥i_≫1. To find these exceptional cases, the *Wind* data set [*Acuña et al.*, 1995] was used, for which 20 years of data are now available. Figure 1 shows the distribution of *β*_⊥i_ measured in the free solar wind during the years 1994–2010 (2.16 × 10^6^ data points). The proton densities and temperatures from the SWE instrument [*Ogilvie et al.*, 1995], found by the fitting technique of *Maruca and Kasper* [2013], were used, along with the magnetic field from the MFI instrument [*Lepping et al.*, 1995] using the calibration of *Koval and Szabo* [2013]. Cases of *β*_⊥i_<0.1 and *β*_⊥i_#x003E;10 occur rarely, making up 5.3% and 0.46% of the data set, respectively.

The data set was searched for low *β*_⊥i_ periods longer than 30 min and high *β*_⊥i_ periods longer than 15 min. Thirty-six low *β*_⊥i_ and 19 high *β*_⊥i_ intervals were selected, which have mean *β*_⊥i_ values within the blue shaded regions in Figure 1. The low *β*_⊥i_ intervals cover the range of solar wind speeds 300–770 km s^−1^ and are within interplanetary coronal mass ejections (ICMEs): the majority (31) coincides with events on the ICME list of *Jian et al.* [2006], and the others also display some of the characteristic signatures. This is expected, since ICMEs make up much of the low *β*_⊥i_ solar wind. While the large-scale properties of ICMEs are quite different to the rest of the solar wind, we do not expect the physics at kinetic scales to significantly differ, meaning that these intervals can be used to study the ion break scale of the magnetic spectrum. The high *β*_⊥i_ intervals cover a range of speeds 290–510 km s^−1^ and are due mainly to short periods of low magnetic field strength, which also have a higher than average density. At high *β*, the solar wind can easily become unstable, and signatures of mirror modes [*Winterhalter et al.*, 1994; *Russell et al.*, 2009; *Bale et al.*, 2009] are present in some of these intervals.

**Figure 2:**
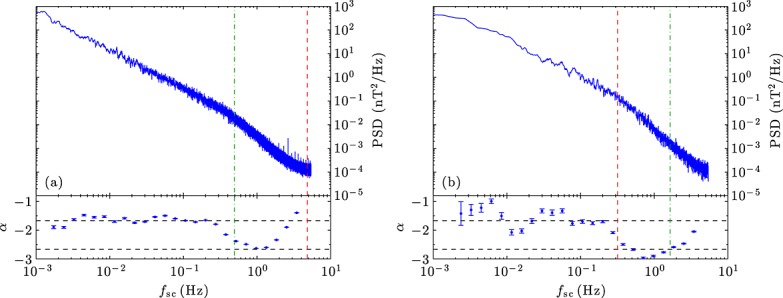
(a) Magnetic power spectrum and local slope *α* for *β*_⊥i_=0.010 (19 January 2005 11:00–24:00). (b) Same for *β*_⊥i_=27 (23 September 2001 22:16–22:41). Frequencies corresponding to *kρ*_i_=1 (red dashed line) and *kd*_i_=1 (green dash-dotted line) are marked. In both cases, the break occurs at the larger of the scales.

Figure 2 shows the magnetic power spectra for a low *β*_⊥i_ and high *β*_⊥i_ interval, calculated from the 92 ms resolution MFI data (with small data gaps linearly interpolated) using the multitaper method [*Percival and Walden*, 1993]. The frequencies corresponding to *kρ*_i_=1 and *kd*_i_=1 are marked assuming the *Taylor* [1938] hypothesis. The individual SWE Faraday cup spectra in these intervals were examined manually to ensure that the automated analysis software correctly determined the ion parameters and a comparison with data from the nearby ACE spacecraft provided additional confirmation. Also shown is the local power law fit *α* in the range 0.58*f*_sc_ to 1.73*f*_sc_. In both cases, *α* is close to −5/3 for frequencies *f*_sc_<0.3Hz, as is well established in this range [*Alexandrova et al.*, 2013; *Bruno and Carbone*, 2013], then becomes smaller before becoming larger once more at the highest frequencies. The flattening of the spectrum at high frequencies is not physical and thought to be due to aliasing of spin tone harmonics [*Koval and Szabo*, 2013]. The steepening at *f*_sc_≈0.5Hz, however, is physical, and is the subject of this letter.

While the observed break frequency *f*_b_ can be found in various ways, here we define it to be the frequency at which *α* takes a value half way between −5/3 and −8/3. Figure 2 shows that *f*_b_ is very close to the frequency corresponding to *kd*_i_=1 in the low *β*_⊥i_ example and to *kρ*_i_=1 in the high *β*_⊥i_ example. In other words, the break occurs at the larger of the two scales.

To investigate the generality of this finding, spectra were calculated for all of the intervals discussed above. While most display a clear break, some have a more complicated shape or do not significantly steepen, which may be due to instrumental noise and the presence of mirror modes at high *β*_⊥i_. The reason for the lack of a break in several of the low *β*_⊥i_ intervals is not known, but it generally occurs when the relative amplitude of fluctuations *δB*/*B*_0_ is lower than average. The *f*_b_ was measurable in 12 of the high *β*_⊥i_ spectra and 18 of low *β*_⊥i_ spectra, and only these were used in the subsequent analysis. The average plasma parameters for these intervals are given in Table 1 along with their standard deviations; electron temperatures were taken from ground moments of distribution functions measured by the 3DP instrument [*Lin et al.*, 1995].

**Table 1:** Mean Interval Parameters

	*β*_⊥i_≪1	*β*_⊥i_≫1
*B* (nT)	13.3 ± 4.1	1.14 ± 0.35
*n*_i_ (cm^−3^)	3.9 ± 2.9	17.1 ± 7.1
*v*_sw_ (km s^−1^)	460 ± 120	389 ± 49
*T*_i_ (eV)	1.64 ± 0.86	4.8 ± 2.9
*T*_e_ (eV)	12.5 ± 4.0	8.6 ± 1.7
*f*_b_ (Hz)	0.58 ± 0.19	0.223 ± 0.068
(*δB*/*B*_0_)_b_	0.0229 ± 0.0077	0.349 ± 0.096
sin(*θ*_Bv_)	0.82 ± 0.12	0.68 ± 0.23
*β*_i_	0.0122 ± 0.0059	23.2 ± 7.4
*β*_e_	0.111 ± 0.054	51 ± 28

**Figure 3:**
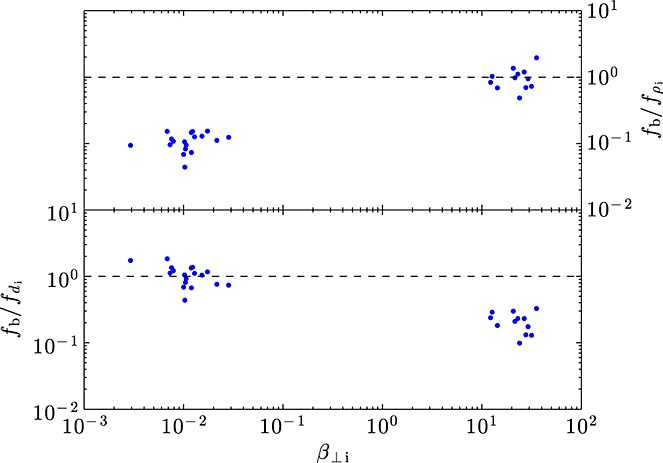
Ratio of measured break frequency *f*_b_ to the frequency corresponding to (top) *kρ*_i_=1 and (bottom) *kd*_i_=1, as a function of *β*_⊥i_ for all intervals in which a break was measurable.

Figure 3 shows the *β*_⊥i_ dependence of the measured break frequency normalized to the frequencies corresponding to the ion gyroscale and ion inertial length, 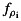
=*v*_sw_/(2*πρ*_i_) and 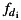
=*v*_sw_/(2*πd*_i_), where *v*_sw_ is the solar wind speed. It is clear that for low *β*_⊥i_ the data points cluster around 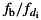
≈1 but are significantly below 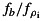
≈1 and at high *β*_⊥i_ the reverse is true. It has been pointed out [e.g., *Leamon et al.*; *Bourouaine et al.*] that if the turbulence is anisotropic in the sense *k*_⊥_≫*k*_∥_, as it is thought to be at kinetic scales [*Chen et al.*], then an additional factor of sin(*θ*_Bv_), where *θ*_Bv_ is the angle between the mean magnetic field and the solar wind direction, should be included in the definition of 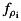 and 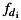. From Table 1 it can be seen that on average this would make at most a factor of 1.5 difference, so the above result would not be affected. Therefore, it appears generally true that the break from a 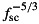
spectrum of magnetic fluctuations occurs at the larger of the ion gyroradius and ion inertial length. This is the main result of this letter.

## 3. Possible Explanations

### 3.1. Alfvén Wave Dispersion

The ion-scale spectral break is often attributed to the scale at which the Alfvénic turbulence, thought to exist above the ion scales [*Alexandrova et al.*, 2013; *Bruno and Carbone*, 2013], becomes dispersive at the transition to kinetic Alfvén turbulence, thought to exist at smaller scales [*Podesta*, 2013; *Chen et al.*, 2013a]. While the extent to which linear theory applies to strong turbulence in the solar wind is an open question, the scale at which Alfvén waves become dispersive can be derived from linear kinetic theory at both low and high *β*_i_. It is assumed, based on observations [e.g., *Chen et al.*, 2010a, 2010b], that the turbulence is anisotropic *k*_⊥_≫*k*_∥_ at kinetic scales and each species takes an equilibrium isotropic Maxwellian distribution for simplicity.

For *β*_i_≫1, *kρ*_i_ dispersion corrections dominate *kd*_i_ corrections, so the latter can be neglected, leading to Alfvén waves with frequency *ω* ≪ *Ω*_i_ and *ω* ≪ *k*_∥_*v*_th,i_. For *k*_∥_*ρ*_i_≪1 and *k*_⊥_*ρ*_i_≪1, the dispersion relation, with the *kρ*_i_ terms retained, becomes


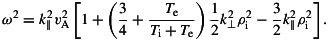
1

Since *T*_i_∼*T*_e_ for the *β*_⊥i_≫1 intervals (Table 1) and we are assuming *k*_⊥_≫*k*_∥_, equation 1 indicates that the break should occur at *k*_⊥_*ρ*_i_∼1. This agrees with the measurements in section 2 which show the break at the ion gyroscale at high *β*_⊥i_.

Similarly, the predicted break can be found for *β*_i_≪1. Dispersion corrections related to *ρ*_i_ can now be neglected giving *k*_∥_*v*_th,i_≪*ω* ≪ *k*_∥_*v*_th,e_. This is different to previous studies [e.g., *Lysak and Lotko*, 1996; *Hollweg*, 1999], which have assumed *ω* ≪ *Ω*_i_ and have therefore neglected *d*_i_ corrections. The dispersion relation can be obtained if it is assumed that 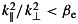. Since *β*_e_≈0.11 in the low *β*_⊥i_ intervals (Table 1), this would require wave vector angles *θ*_kB_>71.6°, which is typically satisfied at these scales [*Chen et al.*, 2010a, 2010b]. In this case, the dispersion relation can be shown to reduce to the Alfvén wave 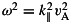 for *k*_⊥_*d*_i_≪1 and the kinetic Alfvén wave 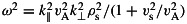
for *k*_⊥_*d*_i_≫1. The break can be estimated to be where these meet, which occurs at


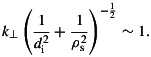
2

This result holds for *T*_i_≪*T*_e_ and corresponds to *k*_⊥_*ρ*_s_∼1if *β*_e_≪1 and *k*_⊥_*d*_i_∼1 if *β*_e_≫1, in agreement with the Hall Reduced MHD dispersion relation [*Schekochihin et al.*, 2009]. This scale, however, does not match the measured break in the *β*_⊥i_≪1 intervals: on average, the frequency corresponding to this scale is 4.8 times larger than *f*_b_ (it is dominated by *ρ*_s_ rather than *d*_i_ since *β*_e_≪1). For *d*_i_ to be relevant for Alfvén wave dispersion at *β*_i_≪1 would require either *β*_e_≫1, which is not the case (Table 1), or a large *k*_∥_ component to the turbulence, which is not generally observed, either in the free solar wind [*Chen et al.*, 2010a, 2010b] or within ICMEs [*Leamon et al.*, 1998b].

### 3.2. Cyclotron Damping

It has been suggested [e.g., *Leamon et al.*, 1998a, *Bruno and Trenchi*, 2014] that the break in the spectrum could be caused by the damping of energy at the ion cyclotron resonance. The condition for this resonance is *ω* − **k**·**v** =± *Ω*_i_, where **v** is the particle velocity, so assuming parallel Alfvén waves, 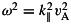, the resonance occurs at *k*_c_=*Ω*_i_/(*v*_A_+*v*_th,i_), where the thermal speed has been used for the particle velocity [*Leamon et al.*, 1998a]. This can also be written as *k*_c_=(*d*_i_+*ρ*_i_)^−1^, meaning that the break would occur at the larger of *ρ*_i_ and *d*_i_, in agreement with the results of section 2. However, since the turbulence is measured to be predominantly anisotropic *k*_⊥_≫*k*_∥_ at these scales [*Chen et al.*, 2010a, 2010b], it is expected to remain low frequency so that cyclotron damping is not efficient at removing energy from the electromagnetic fluctuations [*Quataert*, 1998; *Schekochihin et al.*, 2009]. While such resonances can be broadened in strong turbulence [*Quataert*, 1998; *Lehe et al.*, 2009; *Lynn et al.*, 2012], this is only expected to be an order unity effect. If turbulence within ICMEs is significantly less anisotropic, the cyclotron resonance may become significant; however, *Leamon et al.* [1998b] found it to be more anisotropic at kinetic scales within ICMEs. Therefore, while the cyclotron resonant scale *k*_c_ matches the observed break *f*_b_ at both low and high *β*_⊥i_, it is not consistent with the generally observed anisotropy of the turbulence.

### 3.3. Electron Landau Damping

While Alfvén wave dispersion cannot account for the low *β*_⊥i_ break and the cyclotron resonance is not thought to be important, could Landau damping explain it? The Landau resonance occurs when the phase speed of a wave matches the particle velocity, so can lead to damping even if the fluctuations are low frequency. *Leamon et al.* [1999] suggested that electron Landau damping could lead to the observed break at *β*_i_∼1, and *Howes et al.* [2008] suggested it to be the cause of the break near *d*_i_ in the *β*_i_≪1 interval of *Smith et al.* [2001]. Using the kinetic Alfvén wave electron Landau damping rate derived in *Boldyrev et al.* [2013], along with the parameters in Table 1 for the *β*_⊥i_≪1 intervals, gives a normalized damping rate *γ*/*ω*_0_≈−0.066at *k*_⊥_*ρ*_s_=1. The damping rate at *k*_⊥_*d*_i_=1 will be smaller than this, so is unlikely to be the cause of the spectral break in the current data set. Calculating the Landau damping numerically and applying this to a model spectrum [*Howes et al.*, 2011] also shows that the break occurs closer to *k*_⊥_*ρ*_i_=1 than to *k*_⊥_*d*_i_=1 (J. M. TenBarge, private communication, 2014). Therefore, electron Landau damping also appears unlikely to be the cause of the break at *kd*_i_=1 in the *β*_⊥i_≪1 intervals.

### 3.4. Current Sheets

The break has also been related to the scale of current sheets, which may develop in a turbulent plasma. *Leamon et al.* [2000] reported a good correlation between the break and *d*_i_, assuming *k*_⊥_≫*k*_∥_, and concluded that a significant fraction of the dissipation occurs in reconnecting current sheets, thought to have thickness *d*_i_. *Vasquez et al.* [2007] examined the widths of current sheets in the solar wind and found that while there is significant variability, for *β*_i_<0.1 the width scales better with *d*_i_ and for *β*_i_>4 it scales better with *ρ*_i_. This would agree with the results of section 2 in which the break was found to occur at the larger of these scales, although a causal relationship is not necessarily implied. On the other hand, simulations and laboratory measurements of reconnection with a large guide field [*Cassak et al.*, 2007; *Egedal et al.*, 2007], appropriate for the small amplitude turbulent fluctuations at low *β*_i_, show a sudden onset of reconnection when the current sheet thickness becomes *ρ*_s_, where two-fluid effects become important, rather than *d*_i_. As shown in section 3.1, the frequency corresponding to *kρ*_s_=1 is not close to the measured break frequency for *β*_⊥i_≪1.

## 4. Discussion

We have examined the ion scale break frequency of solar wind turbulence during intervals of *β*_⊥i_≪1 and *β*_⊥i_≫1. While these cases are not typical for the solar wind at 1 AU, they enable the predicted break scales to be measurably different, so that distinguishing between them is physically meaningful. The results of the analysis are summarized in Table 2. The average ratio of the measured break frequency *f*_b_ to the theoretical break frequency for each scale *f*_*x*_ is given, which is closest to unity in the *β*_⊥i_≪1 intervals for *d*_i_ and in the *β*_⊥i_≫1 intervals for *ρ*_i_. The dispersive scale for low *β*_i_ Alfvén waves does not fit the observations and neither does the ion gyrofrequency *Ω*_i_/(2*π*). While the cyclotron resonant wave number does fit the observations at both high and low *β*_⊥i_ within errors, it is not consistent with the observed anisotropy of the turbulence, as discussed in section 3.2. The break is also not at a fixed value of *f*_sc_: the mean break frequency is *f*_b_=0.58Hz in the low *β*_⊥i_ intervals and *f*_b_=0.22Hz in the high *β*_⊥i_ intervals, a factor of 2.6 different. The linear correlation coefficients *r* between *f*_b_ and *f*_*x*_, along with their 95% confidence intervals, are also given in Table 2. The uncertainties are large enough that a meaningful distinction based on the correlation coefficients is not possible. Inclusion of the sin(*θ*_Bv_) factor was not found to significantly alter the correlations.

**Table 2:** Comparison Between Measured Break Frequency and Theoretical Values

	*f*_b_/*f*_*x*_		*r*	
Scale, *x*	*β*_⊥i_≪1	*β*_⊥i_≫1	*β*_⊥i_≪1	*β*_⊥i_≫1
*ρ*_i_	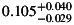	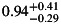	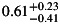	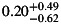
*d*_i_	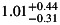	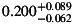	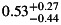	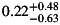
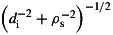	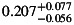	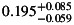	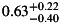	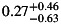
*k*_c_	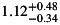	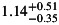	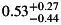	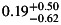
*Ω*_i_/(2*π*)	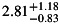	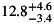	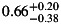	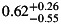

While the break at *ρ*_i_ in the *β*_⊥i_≫1 intervals is consistent with the Alfvén wave dispersion scale, the break at *d*_i_ in the *β*_⊥i_≪1 intervals is not consistent with any of the explanations in section 3, assuming that the fluctuations are anisotropic *k*_⊥_>*k*_∥_. This leaves the possibilities that either the turbulence has a significant *k*_∥_ component in the *β*_⊥i_≪1 intervals, or the break is related to a nonlinear process, such as anomalous resistivity, which may manifest in a plasma with *β*_i_≪*β*_e_<1 (S. Boldyrev et al., manuscript in preparation, 2014). The lack of a break in some of the low *β*_⊥i_ intervals with small *δB*/*B*_0_ is consistent with a nonlinear process. Further studies are required to investigate these possibilities.

As well as in the solar wind, identifying the scale associated with the spectral break is important for understanding turbulence and dissipation in other plasma environments. For example, turbulence is thought to heat the interstellar medium (ISM), in which density fluctuations are well measured but magnetic fluctuations are not [e.g., *Haverkorn and Spangler*, 2013]. The spectral break calculated from radio observations has been used to determine which phase of the ISM is responsible for the observed turbulence [*Spangler and Gwinn*, 1990], so knowing the scale at which the break occurs is important. *Spangler and Gwinn* [1990] assumed the break to be at the larger of *ρ*_i_ and *d*_i_, which is consistent with the results of this letter. Remote measurements of density fluctuations in the solar corona, a low *β*_i_ environment, also show a steepening in the spectrum around the ion inertial length [*Coles and Harmon*, 1989; *Harmon*, 1989; *Harmon and Coles*, 2005] along with a flattening at slightly larger scales that is consistent with the increased compressibility of kinetic Alfvén turbulence [*Harmon*, 1989; *Hollweg*, 1999; *Harmon and Coles*, 2005; *Chandran et al.*, 2009; *Chen et al.*, 2012, 2013b]. The upcoming Solar Probe Plus mission will enable further investigation of these features with in situ measurements of the turbulent fields in the corona.

## Key Points

- Ion-scale spectral break measured in solar wind with very high and low beta
- Break occurs at the larger of the ion gyroradius and ion inertial length
- Results compared to various theoretical expectations

## APPENDIX

### Appendix A: Definitions

The ion gyroradius is defined as *ρ*_i_=*v*_th⊥i_/*Ω*_i_, where  is the perpendicular ion thermal speed, *Ω*_i_=*q*_i_*B*/*m*_i_ is the ion gyrofrequency, *T*_⊥i_ is the perpendicular ion temperature, *m*_i_ is the ion mass, *q*_i_ is the ion charge, and *B* is the magnetic field strength. The ion inertial length is defined as *d*_i_=*c*/*ω*_pi_, where  is the ion plasma frequency, and *n*_i_ is the ion number density. This can also be written *d*_i_=*v*_A_/*Ω*_i_, where  is the Alfvén speed and *ρ*is the total mass density. The ratio of ion thermal pressure to magnetic pressure is *β*_i_=*n*_i_*k*_B_*T*_i_/(*B*^2^/2*μ*_0_). The sound gyroradius is defined as *ρ*_s_=*v*_s_/*Ω*_i_, where is the ion acoustic speed.
